# Laser-Ablated ZnO Nanoparticles and Their Photocatalytic Activity toward Organic Pollutants

**DOI:** 10.3390/ma11071127

**Published:** 2018-07-03

**Authors:** Neli Mintcheva, Ali A. Aljulaih, Wilfried Wunderlich, Sergei A. Kulinich, Satoru Iwamori

**Affiliations:** 1 Institute of Innovative Science and Technology, Tokai University, Hiratsuka, Kanagawa 259-1292, Japan; ali.aljulaih@gmail.com; 2 Department of Chemistry, University of Mining and Geology, Sofia 1700, Bulgaria; 3 Department of Materials Science, Tokai University, Hiratsuka, Kanagawa 259-1292, Japan; wi-wunder@rocketmail.com; 4 Research Institute of Science and Technology, Tokai University, Hiratsuka, Kanagawa 259-1292, Japan; iwamori@tokai-u.jp

**Keywords:** ZnO, laser ablation in liquid, nanospheres, nanorods, photocatalysis

## Abstract

This work aimed to prepare nanostructures of ZnO with various lasers, testing them as photocatalysts, and comparing their morphology and activity in the degradation of organic pollutants in aqueous media. ZnO nanospheres (ns-ZnO) and ZnO nanorods (ms-ZnO) were prepared via the laser ablation of a Zn metal plate in water using nanosecond- and millisecond-pulsed lasers, respectively. The obtained materials were characterized using a set of optical, structural, and surface-science techniques, such as UV-vis spectroscopy, X-ray diffraction (XRD), transmission electron microscopy (TEM), and X-ray photoelectron spectroscopy (XPS). Under visible-light irradiation, both nanostructures were found to be catalytically active toward the oxidation of methylene blue, which was used as a model compound. The ZnO nanorods fabricated with the millisecond laser showed better photocatalytic performance than their spherically shaped counterparts obtained by means of the nanosecond laser, which could be assigned to a larger number of defects on the ms-ZnO surface.

## 1. Introduction

Among various “top-down” approaches for the preparation of nanoparticles (NPs), laser ablation in liquid (LAL) is a sustainable, easy-to-use, and efficient laboratory method for the production of diverse nanomaterials with different chemistry (mainly metal oxides, sulfides, carbides, and metals), and various morphologies and shapes [1,2,3,4,5,6,7,8]. In the last decade, a rapidly growing interest in the laser-assisted synthesis of colloids was observed, which is explained by numerous features associated with LAL, such as reduced amounts (and thus, cost) of reagents and solvents used as precursors, ease of use, highly safe working environment, relatively fast procedures, and so on [1,8,9]. A simple experimental set-up is typically applied in this approach, where a laser beam is focused on a metal (or more complex) target immersed in a small volume of liquid to ablate the target, producing plasma, vapor, or molten-metal drops that will further quench and/or react with the liquid to form NPs with different structures and compositions [1,2,3,4,5,6,7,8,9,10,11,12]. As a typical example, ZnO NPs with different morphology were reported by several groups to be generated via ablating a zinc metal plate in water (or some other solvents) using different types of lasers [2,3,5,6,7,10,11,12,13,14,15,16,17,18,19,20]. 

As one of the most investigated semiconductors, ZnO was fabricated via LAL using different pulsed lasers. Ablation of Zn metal using millisecond-long pulses in water was reported to give ZnO nanorods with different aspect ratios depending on pulse width and pulse energy [5], while in ethanol, spherical NPs were produced with the same laser [5]. Under certain conditions, and in well-selected liquid media, hollow ZnO NPs could also be produced using millisecond pulses [2,3]. The most commonly used nanosecond pulsed lasers were applied to produce ZnO NPs with different sizes and photoluminescence (PL) properties [6,7,11,12,13,16,17,20]. Kim et al. found significant size and shape changes when the laser wavelength decreased from 1064 nm to 355 nm [14]. The effect of medium pressure on the sizes and PL properties of the produced ZnO NPs was also studied [7,20]. In addition, LAL is an effective way of producing doped ZnO. As examples, Qin et al. [15] produced Cu-doped ZnO with a strictly controlled dopant concentration and a tunable emission wavelength, while Mg-doped ZnO NPs with a blue-shifted band gap were generated using a femtosecond-pulsed laser [16]. Dual N- and Ag-doped ZnO materials were produced via consecutive laser ablation of Zn and Ag in NH_4_NO_3_ solution [17]. ZnO NPs, ablated using a picosecond-pulsed laser in tetrahydrofuran, were found covered with a polyurethane layer, and thus, appeared green PL [18]. 

As already mentioned, the PL properties of laser-generated ZnO NPs were extensively investigated [7,11,12,13,20]. More recently, the gas-sensing properties of ZnO prepared by ablation with nanosecond- and millisecond-pulsed lasers were also tested, demonstrating promising room-temperature detection of ethanol with good selectivity and detection limits on the order of 50 ppm [10]. Although various LAL-generated semiconductor nanostructures were previously reported to demonstrate attractive photocatalytic properties [1,8,21,22], surprisingly little was done on ZnO nanostructures prepared by lasers in the liquid phase [19]. Abbas and Bidin first prepared ZnO NPs using a nanosecond-pulsed laser, after which the prepared colloid was modified through hydrothermal treatment at different pH and for different time periods, achieving materials with different morphologies. The hydrothermally modified materials revealed that an enhanced surface area and an increase in defects were the main factors for high photocatalytic activity toward the degradation of methylene blue (MB) under sunlight irradiation [19]. Work on the photocatalytical performance of as-prepared ZnO NPs generated using LAL is unreported as of now, which was one of the motivations stimulating the presented study.

LAL is well known to provide very high temperatures and temperature gradients inside and around the ablation zone, as well as extremely high quenching rates between pulses, often leading to unique NP morphologies, metastable phases, and nanostructures with defect-rich surfaces [1,2,3,8,20,21,22]. Because surface defects are highly likely sites for the gas-sensing and catalytic activities of materials, it is natural to expect that LAL is an efficient technique of producing NPs with high catalytic activities toward different pollutants. Therefore, in this work, we aimed to prepare ZnO nanomaterials with different morphology (nanorods and nanospheres), and to evaluate their photocatalytic performance toward methylene blue (MB). Lasers with millisecond- and nanosecond-long pulses were used, which provided ZnO nanostructures with rod-shaped and spherical-shaped morphology, respectively. The produced materials were then characterized using a set of structural and chemical analysis techniques, after which their ability to degrade MB in the aqueous phase and under visible-light irradiation was evaluated. 

## 2. Materials and Methods 

Both samples were prepared using Nd:YAG lasers with a wavelength of 1064 nm. In both cases, the laser beam was focused on metal targets with spot sizes of 150–200 µm. The target was a Zn metal plate, which was placed vertically into a quartz cuvette with dimensions of 30 mm × 30 mm × 50 mm, and a wall thickness of 2 mm. Neat deionized water (15 mL) with no surfactants was used as a medium, which was agitated by a magnetic stirrer during the experiments. The procedures were previously reported in more detail elsewhere [5,10,20,23,24]. 

In accordance with our previous results [5,10], the rod-shaped ZnO nanomaterial (hereafter denoted as ms-ZnO) was prepared using a millisecond-pulsed laser, with the following applied parameters: peak pulse power of 5 kW, pulse width of 1.0 ms (which corresponded to a pulse energy of 5.0 J), pulse frequency of 5 Hz, and an ablation time of 30 min. The second sample (denoted as ns-ZnO hereafter) was prepared by means of a nanosecond-pulsed laser, which was previously reported to generate spherical NPs [7,10,20]. The experimental set-ups used were similar for both samples. The laser beam parameters used for this sample were as follows: a pulse energy of 120 mJ/pulse, a pulse width of 7 ns, and a repetition rate of 10 Hz. The treatment time was also set at 30 min.

The freshly prepared colloids were first evaluated (in liquid phase) with UV-vis spectroscopy, after which they were centrifuged and rinsed with water. Finally, after redispersion, the products were adjusted to a volume of ~1.5 mL and drop-cast onto glass plates for further annealing at 400 °C for 2 h (for catalytic test), or onto Si wafers (for phase and chemical analyses). For transmission electron microscopy (TEM), one drop of each colloid was placed onto a Cu grid, and dried in air. For the photocatalytic tests, the samples were used both as prepared and after annealing. The concentrated as-prepared suspensions were directly added to the methylene blue (MB) solution, while their annealed counterparts were immersed into aqueous MB as coatings drop-cast onto glass plates. 

The photocatalytic activity of both laser-ablated samples was evaluated via the photo-degradation of MB under visible-light irradiation. The catalytic tests were carried out using 50 mL of aqueous MB with a concentration 8 × 10^−6^ mol/L, and 6 mg of photocatalyst. The mixture was irradiated with a 1000-W tungsten-halogen lamp for 210 min. Prior to irradiation, the reaction mixture was kept in the dark for 30 min to reach an adsorption–desorption equilibrium between the dye molecules and the catalyst surface. The MB decomposition process was monitored via absorption changes of its characteristic band at a 664-nm wavelength using UV-vis spectroscopy. After each 30 min interval, an aliquot of 4 mL was pipetted, and its UV-vis spectrum was recorded, before the solution was returned back to the reactor. 

The prepared nanomaterials were characterized using UV-vis spectroscopy (UV-2450 model from Shimadzu, Kyoto, Japan, X-ray diffractometry (XRD; D8 Discover from Bruker, Yokohama, Japan), transmission electron microscopy (HF-2200 tool from Hitachi, Tokyo, Japan), and X-ray photoelectron spectroscopy (XPS, Quantum 2000, ULVAC-PHI, Inc., Chigasaki, Japan). The photocatalytic reactions were carried out with both as-prepared and annealed samples of ms-ZnO generated using a millisecond laser (ML-2150A from Miyachi Co. Ltd., Isehara, Japan), and ns-ZnO obtained by means of a nanosecond laser (Surelite SL I-10 from Continuum Co., San Jose, CA, USA). 

## 3. Results and Discussion

**Figure 1:**
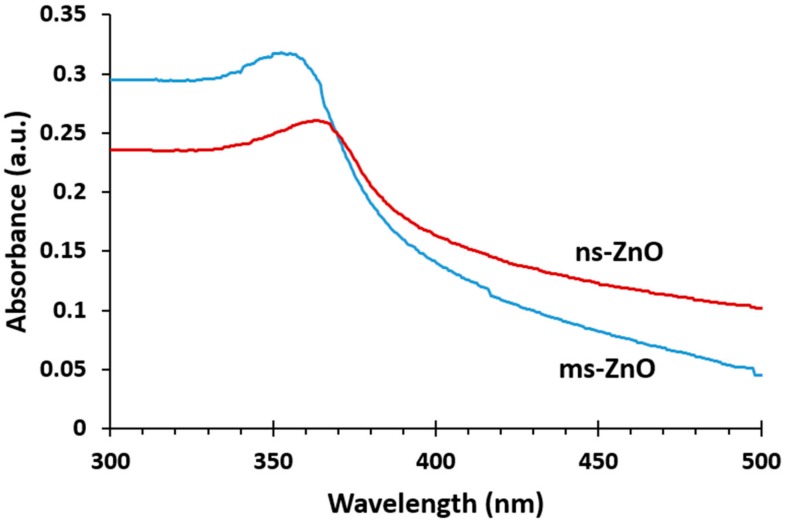
UV-vis absorption spectra of as-prepared ZnO nanosphere (ns-ZnO) and ZnO nanorod (ms-ZnO) colloids in water.

Figure 1 presents the UV-vis absorption spectra of both as-prepared ZnO colloids. The peaks observed at 355 nm and 365 nm for ms-ZnO and ns-ZnO samples, respectively, are characteristic of ZnO NPs. It is well known that the absorption peak of ZnO in the wavelength range of 300–400 nm is due to the intrinsic absorption of ZnO when electrons are promoted from the valence band to the conduction band of the semiconductor [5,25]. A blue shift of the absorption maximum for ms-ZnO in comparison with that of ns-ZnO was observed, which probably arose from a decrease in NP size and a larger density of defects in the ms-ZnO. Importantly, no peaks in the range of 270–280 nm were observed, implying there was no inclusion of the metallic Zn phase in the as-prepared NPs [5].

**Figure 2:**
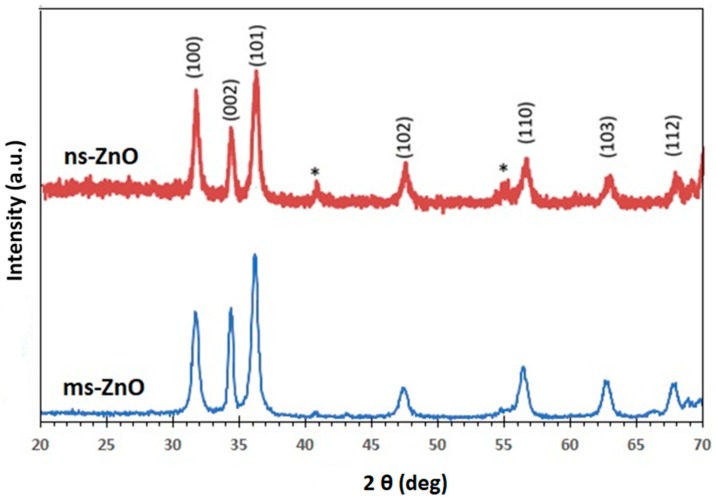
X-ray diffraction (XRD) patterns of ns-ZnO and ms-ZnO samples prepared using nanosecond- and millisecond-pulsed lasers, respectively. Multiple signals due to the Si substrate are marked by asterisks. All signals in both patterns were indexed as hexagonal ZnO.

Figure 2 displays the XRD patterns of ns-ZnO and ms-ZnO samples, following their fresh preparation and being drop-cast onto Si wafers. All peaks were indexed as the hexagonal wurtzite structure of ZnO (JCPDS card no. 36-1451). No traces of other phases were observed, indicating the absence of metallic Zn inclusions, which agreed well with the UV-vis spectra discussed above. This finding is consistent with previously reported results for various laser-produced ZnO NPs [5,6,7,10,11,13,14]. Inclusions of metallic zinc were often observed in laser-generated ZnO NPs produced in ethanol or other organic liquids, as well as in their mixtures with water, implying a lack of enough oxygen during the LAL process in such media [5,20]. It should be noted, however, that based on the wide peaks observed in Figure 2, a certain fraction of amorphous ZnO and/or Zn(OH)_2_ phases cannot be excluded as inclusions into the main phase, in accordance with previous reports on LAL-prepared ZnO NPs [6,7,14,20]. Upon annealing, the samples demonstrated very similar XRD patterns; thus, they are not shown here for the purpose of brevity. 

**Figure 3:**
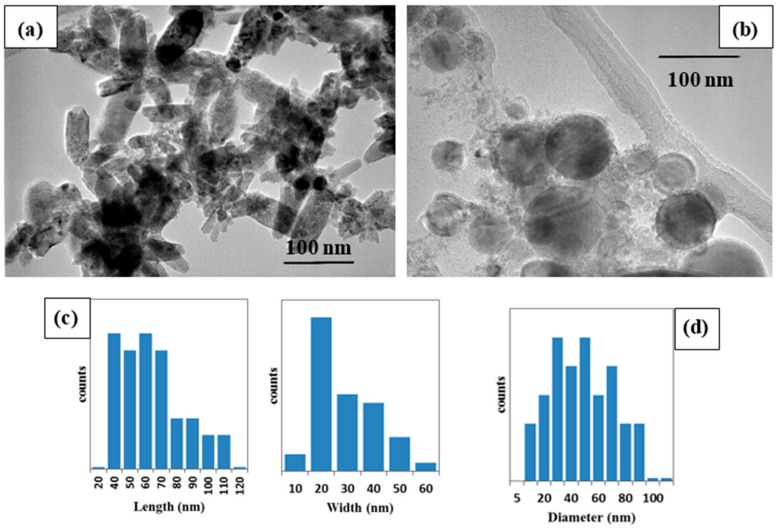
Transmission electron microscopy (TEM) images of (**a**) ms-ZnO nanoparticles (NPs; nanorod-shaped) prepared using the millisecond-pulsed laser, and (**b**) ns-ZnO NPs (sphere-shaped) prepared using the nanosecond-pulsed laser; (**c**) size distribution (length and width) of the ZnO nanorods; (**d**) size distribution of the ZnO nanospheres.

The morphologies of the two ZnO samples were confirmed using TEM analysis (Figure 3). The ms-ZnO NPs prepared using the millisecond-pulsed laser were rod-shaped (Figure 3a), with lengths in the range of 40–110 nm, and an average width of 30 nm (Figure 3c). Their shape and sizes are in good agreement with previously reported rod-shaped ZnO NPs which were also produced by LAL [5,6]. Tunable ZnO nanorods were achieved upon changing the pulse width and peak power of the millisecond laser. The growth of nanorod-like nanostructures was explained by (i) the high surface energy of ZnO NPs which led to their agglomeration in the absence of a surfactant, (ii) the temperature rise of the liquid caused by long-pulse irradiation, and (iii) the use of neat water, i.e., without any additives [5]. In the presented work, during the preparation of the ms-ZnO and ns-ZnO samples, their media were found to have temperatures of 80–83 °C and 25–27 °C, respectively. This explains the rod-like morphology observed in Figure 3a, and agrees with previously reported results [5,6]. The ns-ZnO sample produced with the nanosecond-pulsed laser is seen in Figure 3b to be composed of spherical NPs with sizes ranging from about 10 nm to 90 nm (Figure 3d). Similar spherical ZnO nanostructures were previously observed as the product of Zn ablation in water by means of various lasers with ns-long pulses [6,7,10,11,14].

**Figure 4:**
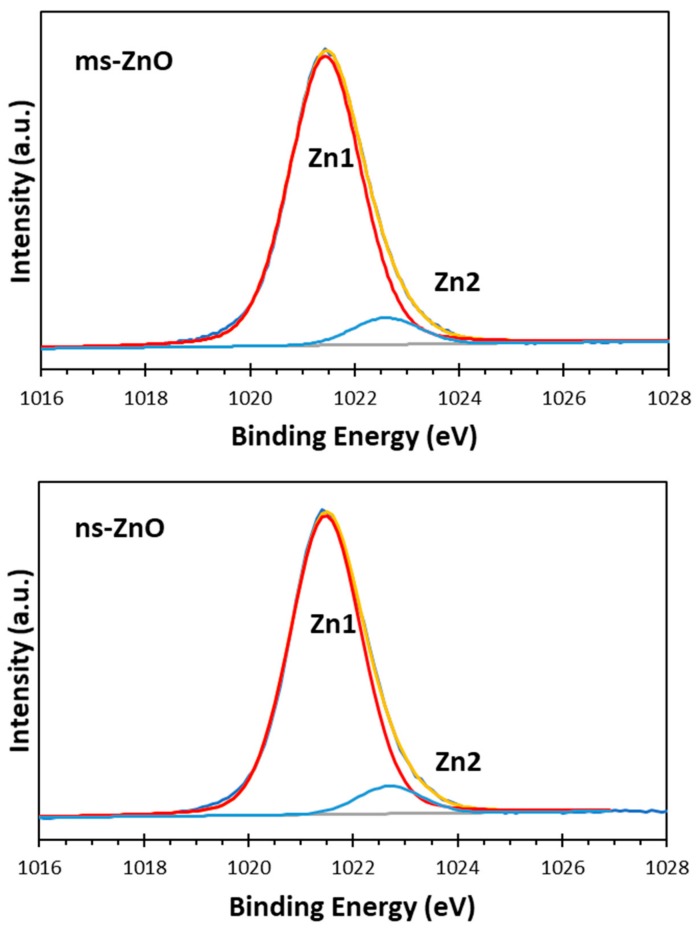
X-ray photoelectron spectroscopy (XPS) Zn 2p_3/2_ spectra of ms-ZnO and ns-ZnO samples curve-fitted with two components corresponding to ZnO (Zn1) and Zn–OH species (Zn2).

**Figure 5:**
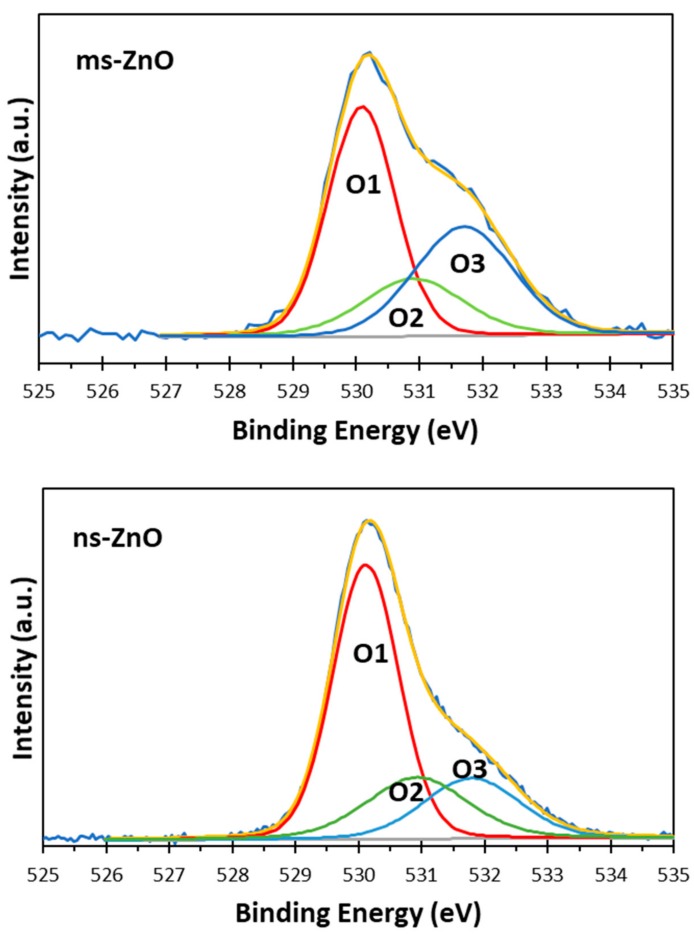
XPS O 1s spectra of ms-ZnO and ns-ZnO samples curve-fitted with three components corresponding to an O^2−^ ion in the wurtzite phase surrounded by Zn atoms in their full tetrahedral geometry of nearest oxygen atoms (O1), surface OH groups bonded to Zn^2+^ (O2), and chemisorbed oxygen, such as O_2_ and adsorbed H_2_O (O3).

The surface chemistry and oxidation states of the zinc and oxygen atoms in the prepared samples were studied using XPS analysis. The XPS Zn 2p spectra of both ns-ZnO and ms-ZnO samples were found to be very similar, with doublets at 1021.5 ± 0.1 eV and 1044.6 ± 0.1 eV, corresponding to Zn 2p_3/2_ and Zn 2p_1/2_, respectively, and with a typical splitting of 23.1 eV [26,27,28]. The deconvolution of Zn 2p_3/2_ peaks for ms-ZnO and ns-ZnO is shown in Figure 4. The components observed at ~1021.5 eV and ~1022.7 eV were assigned to Zn^2+^ ions in a ZnO crystal lattice and to the Zn–OH bond, respectively [5,24,29]. The presence of surface hydroxyl groups was supported by the XPS O 1s spectra of both samples, displayed in Figure 5. The asymmetric peaks in Figure 5 could be curve-fitted by three peaks, denoted as O1, O2, and O3, with binding energies at 530.1 eV, 530.9 eV, and 531.8 eV, respectively. The first one (O1) was assigned to the O^2−^ ions from the wurtzite ZnO lattice, which were surrounded by Zn atoms in their full regular-tetrahedral geometry of nearest oxygen atoms. The peak at 530.9 eV (O2) was ascribed to the surface hydroxyl groups, in agreement with previous reports by others [26,27]. The highest-energy peak at 531.8 eV (O3) was assumed to be due to chemisorbed oxygen whose binding energy is often observed around the same position [28,30]. Altogether, the XPS results agreed well with the XRD results presented in Figure 2, confirming that both samples were ZnO NPs with no metallic Zn inclusions, and with surface Zn–OH species, the latter being consistent with previous works on ZnO NPs prepared via LAL in water [5,11,12,13,14,24]. 

The comparison of peak components in Figure 4 and Figure 5 allows one to draw some conclusions. Firstly, since the peak areas of the Zn1 and Zn2 components, as well as those of O1 and O2, in both samples were similar, it is reasonable to assume that the fractions of both ZnO and Zn–OH species in the surfaces of both samples were comparable. Secondly, because the area of the O3 component in the ms-ZnO sample (Figure 5) was larger, the sample had more oxygen and water molecules chemisorbed onto its surface. It was previously reported by others that such groups detected using XPS are strongly bound to the surface defects on ZnO, and are associated with the presence of oxygen vacancies or defects [25]. Note that after annealing at 400 °C, both samples demonstrated very similar XPS spectra (not shown here for brevity), with only a slightly lower contribution of Zn2, O2, and O3 (responsible for zinc hydroxide and chemisorbed water) components. Importantly, the ms-ZnO sample still exhibited a somewhat larger amount of all the above-mentioned components than the ns-ZnO sample. Therefore, the ms-ZnO sample can be speculated to have a higher density of surface defects, both as prepared and annealed, which would enhance its photocatalytic performance. The potential ability of the ms-ZnO to adsorb the dye molecules onto its surface would facilitate the photocatalytic degradation of MB, and increase its photocatalytic activity.

**Figure 6:**
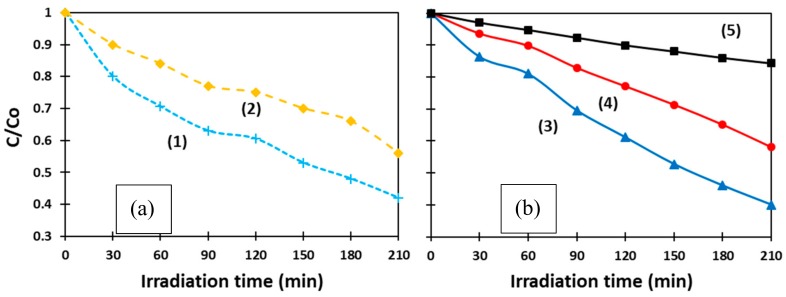
Photodegradation curves of methylene blue (MB) (**a**) in the presence of as-prepared ms-ZnO (1) and ns-ZnO (2); (**b**) in the presence of annealed ms-ZnO (3) and ns-ZnO (4). Curve (5) represents the blank sample.

Among others, the most anticipated potential environmental applications of non-toxic and efficient semiconductor photocatalysts, such as ZnO, TiO_2_, SnO_x_, and the like, are expected to be in the treatment of organic contaminants in water media [31,32]. This stimulated us to investigate the photocatalytic properties of laser-produced ZnO NPs in an aqueous medium under visible-light irradiation. Following standard protocols [19,25,33], UV-vis spectroscopy was used to monitor time-dependent absorption spectra of MB in the presence of ns-ZnO or ms-ZnO under irradiation. The intensity of the characteristic band of MB at 664 nm was found to gradually decrease over irradiation time, thus revealing MB photodegradation upon contact with ZnO NPs. Figure 6 exhibits how the concentration of MB changed with respect to its initial value (C/C_o_) over time. The curves present the behavior of as-prepared ms-ZnO (1) and ns-ZnO (2) (Figure 6a), as well as annealed ms-ZnO (3) and ns-ZnO (4), and a blank sample (5) (Figure 6b). The latter sample was aqueous MB irradiated without any ZnO material. 

The photocatalytic activity of both samples (ms-ZnO and ns-ZnO) is seen in Figure 6 to be comparable, slightly higher for the ms-laser-prepared sample, showing no strong effect of heat treatment (400 °C, 2 h). In Figure 6a, curves (1) and (2), representing the behavior of both ms-ZnO and ns-ZnO samples in the form of as-prepared colloids, exhibit a quicker decrease in MB concentration over the first 90 min. Then, between 90 min and 120 min, MB degradation slowed down, which is seen as the stabilization in C/C_o_ values for both curves (1) and (2) in Figure 6a. On the other hand, the degradation rate of MB over the annealed samples (curves (3) and (4) in Figure 6b) seems monotonous and steady, and both curves in Figure 6b are almost linear. 

This difference between the curves in Figure 6a,b is believed to be due to MB molecule adsorption onto the NP surface, which was more intense in the case of fresh non-annealed NPs having a higher contact area. Although all four samples in Figure 6 were kept in the dark for 30 min prior to the photocatalytic test, we assume this was not enough to establish an adsorption–desorption equilibrium over the as-prepared ZnO NPs. Such an equilibrium was only reached by ~120 min, after which the MB concentration decreased monotonously, eventually reaching very similar C/C_o_ values after 210 min to those in Figure 6b. The annealed samples drop-cast onto glass slides were believed to reach equilibrium with adsorbed MB molecules much faster, resulting in their MB degradation curves in Figure 6b being almost linear.

It can be seen that the photocatalytic performance of the ms-ZnO sample was slightly higher, as both its as-prepared and annealed NPs (curves (1) and (3)) decomposed MB faster than those prepared using the ns-pulsed laser (curves (2) and (4)). Interestingly, no noticeable effect of heat treatment (400 °C, 2 h) was found, as both curves (1) and (3) exhibited comparable photocatalytic activity of ms-ZnO tested as fresh NPs and an annealed coating, respectively. A very similar trend was also observed for curves (2) and (4), which represent the ns-ZnO NPs.

At the end of the experiments, after 210 min irradiation time, the degradation rates of MB over the annealed catalysts, calculated as (1 − C/C_o_) × 100, were found to be ~60% and ~42% for the ms-ZnO and ns-ZnO samples, respectively (Figure 6b), and very similar values were observed for the fresh NPs (Figure 6a). It should be noted that, in the presented study, the photocatalytic experiments were carried out with relatively small amounts of photocatalyst. More specifically, 6 mg of catalyst was used per 50 mL of 8 × 10^−6^ mol/L MB solution, which implies 6 mg of ZnO per 4 × 10^−7^ mol of MB. For comparison, at least twice the amount of ZnO catalyst, with respect to MB, was used by Zhang et al. (i.e., 250 mg of ZnO per 8 × 10^−6^ mol of MB) [25]. This explains the relatively moderate degradation of MB achieved in our experiments after 210 min, as shown in Figure 6. 

The generally accepted mechanism of photocatalysis is known to have several stages involving electron transfer [25,33,34,35]. Firstly, if a semiconductor is irradiated by light with an energy equal to or higher than its band gap, an electron from the valence band (VB) gets excited to its conduction band (CB), resulting in an electron–hole pair (e^−^–h^+^). Secondly, the excited electron can be easily accepted by electron acceptors such as adsorbed O_2_, producing a superoxide radical anion (●O_2_^−^). Thirdly, the hole in the valence band can interact with electron donors such as OH^−^ or H_2_O to form a ●OH free radical (or ●OH and H^+^ in case of H_2_O). Finally, the as-formed strong oxidizing agents (●O_2_^−^, ●OH, and h^+^) then readily and effectively take part in the oxidation of organic molecules. When the semiconductor nanocrystal is free of crystal defects, the photogenerated electrons and holes can quickly experience recombination, which drastically reduces its photocatalytic activity. Therefore, if the electron acceptor traps the excited electrons in its CB, this is expected to suppress their recombination with photoinduced holes. Oxygen defects on NP surfaces are known to efficiently constrain the e^−^–h^+^ recombination, as the oxygen vacancies can accept photogenerated electrons. 

Based on Figure 5, the sample produced by means of the millisecond-pulsed laser was believed to have more oxygen defects, explaining its enhanced photocatalytic activity when compared with the sample produced using the nanosecond-long pulses (ns-ZnO). Thus far, very few studies revealed the relationship between the structure of ZnO NPs and their photocatalytic properties, suggesting that the presence of defects in such nanocrystals is the main factor for promotion of their photocatalytic activity, as such defects enhance the electron–hole separation and facilitate the interactions with photogenerated carriers [25,33,34,35]. Han et al. found that the photocatalytic efficiency of ZnO NPs with different morphology depended on the chemisorption ability of their exposed crystal planes [36]. Higher gas-sensing and photocatalytic properties were observed for ZnO nanoflakes with Zn-terminated surfaces, allowing the enhanced adsorption of O_2_ and OH^−^ species [36]. Jeong et al. found that ZnO nanorods produced via the solvothermal treatment of zinc acetate were more active in the photocatalytic decomposition of MB and phenol, with both processes initiated by hydroxyl radicals and involving single-electron transfer [37]. Furthermore, they reported that ZnO plates generated from zinc chloride under the same conditions were more efficient for the production of H_2_ and H_2_O_2_, both reactions involving two-electron transfers [37]. Thus, plotting links between the morphology of ZnO NPs, their defects, and photocatalytic behavior is becoming an active research topic, in which LAL-generated ZnO nanomaterials can be of great use, as the technique can provide such nanomaterials with varied sizes, phase compositions, and defects [1,5,6,7,11,12,20].

## 4. Conclusions

This study compared ZnO nanomaterials prepared via ablating Zn metal plates in water by means of nanosecond- and millisecond-long laser pulses. ZnO nanomaterials with spherical and nanorod morphologies were obtained, respectively. The prepared materials were characterized, and then applied to the photodegradation of methylene blue in aqueous media under visible-light irradiation. The nanorods generated using the millisecond laser had more chemisorbed water, oxygen molecules, and oxygen defects, which was believed to be the reason why they showed somewhat higher photocatalytic activity when compared with their spherical ZnO counterparts prepared using the nanosecond-pulsed laser. The work, thus, demonstrates that ZnO-based nanomaterials with different shapes and surface defects can be prepared by means of laser ablation in liquid media. Such laser-produced nanostructures have defect-rich surfaces, which appears to be promising for their enhanced photocatalytic performance. Varying the laser parameters and liquid media is seen, therefore, as a simple and attractive approach to achieving catalysts with tuned properties.
